# Monitoring of Farm-Level Antimicrobial Use to Guide Stewardship: Overview of Existing Systems and Analysis of Key Components and Processes

**DOI:** 10.3389/fvets.2020.00540

**Published:** 2020-08-21

**Authors:** Pim Sanders, Wannes Vanderhaeghen, Mette Fertner, Klemens Fuchs, Walter Obritzhauser, Agnes Agunos, Carolee Carson, Birgitte Borck Høg, Vibe Dalhoff Andersen, Claire Chauvin, Anne Hémonic, Annemarie Käsbohrer, Roswitha Merle, Giovanni L. Alborali, Federico Scali, Katharina D. C. Stärk, Cedric Muentener, Ingeborg van Geijlswijk, Fraser Broadfoot, Lucie Pokludová, Clair L. Firth, Luís P. Carmo, Edgar Garcia Manzanilla, Laura Jensen, Marie Sjölund, Jorge Pinto Ferreira, Stacey Brown, Dick Heederik, Jeroen Dewulf

**Affiliations:** ^1^The Netherlands Veterinary Medicines Institute (SDa), Utrecht, Netherlands; ^2^Centre of Expertise on Antimicrobial Consumption and Resistance in Animals (AMCRA), Brussels, Belgium; ^3^Department of Veterinary and Animal Sciences, University of Copenhagen, Frederiksberg, Denmark; ^4^Department for Data, Statistics and Risk Assessment, Austrian Agency for Health and Food Safety, Vienna, Austria; ^5^Unit of Veterinary Public Health and Epidemiology, Institute of Food Safety, Food Technology and Veterinary Public Health, University of Veterinary Medicine, Vienna, Austria; ^6^Public Health Agency of Canada, Guelph, ON, Canada; ^7^Division for Risk Assessment and Nutrition, National Food Institute, Technical University of Denmark, Lyngby, Denmark; ^8^Research Group for Genomic Epidemiology, National Food Institute, Technical University of Denmark, Lyngby, Denmark; ^9^Epidemiology, Health and Welfare Unit, French Agency for Food, Environmental and Occupational Health & Safety, Ploufragan, France; ^10^IFIP-Institut du Porc, Domaine de la Motte au Vicomte, Le Rheu, France; ^11^Unit for Epidemiology, Zoonoses and Antimicrobial Resistance, Federal Institute for Risk Assessment (BfR), Berlin, Germany; ^12^Institute for Veterinary Epidemiology and Biostatistics, Freie Universität Berlin, Berlin, Germany; ^13^Istituto Zooprofilattico Sperimentale della Lombardia e Dell'Emilia Romagna, Brescia, Italy; ^14^SAFOSO AG, Bern, Switzerland; ^15^Institute of Veterinary Pharmacology and Toxicology, University of Zurich, Zurich, Switzerland; ^16^Veterinary Medicines Directorate, Addlestone, United Kingdom; ^17^Institute for State Control of Veterinary Biologicals and Medicines, Brno, Czechia; ^18^Vetsuisse Faculty, Veterinary Public Health Institute, University of Bern, Bern, Switzerland; ^19^Moorepark Animal and Grassland Research Center, Teagasc, Irish Agriculture and Food Development Authority, Cork, Ireland; ^20^School Veterinary Medicine, University College Dublin, Dublin, Ireland; ^21^Danish Veterinary and Food Administration, Glostrup, Denmark; ^22^Department of Animal Health and Antimicrobial Strategies, National Veterinary Institute, Uppsala, Sweden; ^23^Veterinary Epidemiology Unit, Faculty of Veterinary Medicine, Ghent University, Merelbeke, Belgium

**Keywords:** antimicrobial use, livestock, overview, indicator, benchmarking, monitoring, antimicrobial stewardship, antimicrobial resistance

## Abstract

The acknowledgment of antimicrobial resistance (AMR) as a major health challenge in humans, animals and plants, has led to increased efforts to reduce antimicrobial use (AMU). To better understand factors influencing AMR and implement and evaluate stewardship measures for reducing AMU, it is important to have sufficiently detailed information on the quantity of AMU, preferably at the level of the user (farmer, veterinarian) and/or prescriber or provider (veterinarian, feed mill). Recently, several countries have established or are developing systems for monitoring AMU in animals. The aim of this publication is to provide an overview of known systems for monitoring AMU at farm-level, with a descriptive analysis of their key components and processes. As of March 2020, 38 active farm-level AMU monitoring systems from 16 countries were identified. These systems differ in many ways, including which data are collected, the type of analyses conducted and their respective output. At the same time, they share key components (data collection, analysis, benchmarking, and reporting), resulting in similar challenges to be faced with similar decisions to be made. Suggestions are provided with respect to the different components and important aspects of various data types and methods are discussed. This overview should provide support for establishing or working with such a system and could lead to a better implementation of stewardship actions and a more uniform communication about and understanding of AMU data at farm-level. Harmonization of methods and processes could lead to an improved comparability of outcomes and less confusion when interpreting results across systems. However, it is important to note that the development of systems also depends on specific local needs, resources and aims.

## Introduction

Antimicrobial resistance (AMR) is acknowledged as one of the main threats to human health worldwide. It is widely recognized that antimicrobial use (AMU) leads to the selection of resistant bacteria (1), and that animals may constitute one of the reservoirs of resistant bacteria and resistance genes (2–4). Recently, an association between the use of certain antimicrobials in animals and the occurrence of AMR in certain clinical isolates from humans has been shown (5–7). Consequently, reducing AMU in both humans and animals is an essential step toward limiting the prevalence of AMR in both humans and animals.

At the end of the previous millennium, the concept of antimicrobial stewardship (AMS) was established as a set of “responsible use” policy measures aimed at combatting AMR in human hospitals (8). AMS programs have since become common tools in human medicine (9). Following an increased focus on “One Health”; which emphasizes the interconnection between human, veterinary and environmental health, the need for more prudent use practices in veterinary medicine has become more widely accepted, i.e., using antimicrobials “only when necessary” and with treatment decisions based on the diagnosis, including pathogen and relevant resistance data (10).

Collection of reliable AMU data is crucial for the establishment of AMS programs and to measure their effectiveness. In veterinary medicine, major steps have been taken in many countries regarding the development and implementation of systems for collecting national sales data of antimicrobial medicinal products. At the European Union (EU)/European Economic Area (EEA) level, these data are collated by the European Surveillance of Veterinary Antimicrobial Consumption (ESVAC) project at the European Medicines Agency (EMA). The latest ESVAC report included sales data from 31 countries (11), having increased from nine countries in the initial ESVAC report (12). Data is provided voluntarily to ESVAC. In the future collecting data on the volume and use of antimicrobials will become mandatory for EU member states (13). The data published in ESVAC reports has been shown to be important for policymaking, including AMS at the national level, such as setting targets for reducing overall sales and, in particular, of critically important antimicrobials (CIAs). However, accurately determining AMU by animal species using sales data is complicated by the fact that VMPs are labeled for use in multiple species and often used off-label in other species. Furthermore, antimicrobial sales quantification typically does not take dosing differences between antimicrobials into consideration. Moreover, availability of reliable AMU data at the level of the end-user and/or prescriber or provider of the medicinal products (farmer, veterinarian, pharmacies, or feed mills), is vital for guiding farm- and/or sector-specific AMU practices (14–16), targeting unnecessary or inappropriate use, encouraging improvements in animal husbandry, disease prevention and control, and enabling detailed risk and trend analyses.

Many countries are at the initial or advanced stages of setting up systems for monitoring AMU at farm-level by animal species in all or some (food-producing) animal species. Setting up such systems involves various challenges to be faced and decisions to be made, for example, how to organize the data collection, the type and detail of the collected data, choice of indicators for reporting results, benchmarking criteria for acceptable or non-acceptable use, etc. The aim of this paper is to describe known farm-level AMU monitoring systems and discuss their key components and processes. This should provide support for establishing or working with a system and lead to a better implementation and evaluation of stewardship measures. Furthermore, it could be a step toward an improved understanding and sharing of knowledge as well as a more uniform communication across systems and countries. This would make it easier to identify and understand the effects of factors influencing AMU, such as animal health status, presence or absence of certain diseases, biosecurity levels, vaccination programs, historically developed practices, cultural differences, etc., and ultimately, AMR.

This paper was written within the framework of the AACTING project (a Global “network on quantification of Antimicrobial consumption in animals at farm-level and Analysis, CommunicaTion and benchmarkING to improve use”), which was funded by the Joint Programming Initiative on Antimicrobial Resistance (JPI-AMR, project number 270610). A detailed overview of the characteristics of the current systems in each country is available on the AACTING website^1^.

## Overview of Existing Systems for AMU Monitoring at Farm-Level

**Figure 1:**
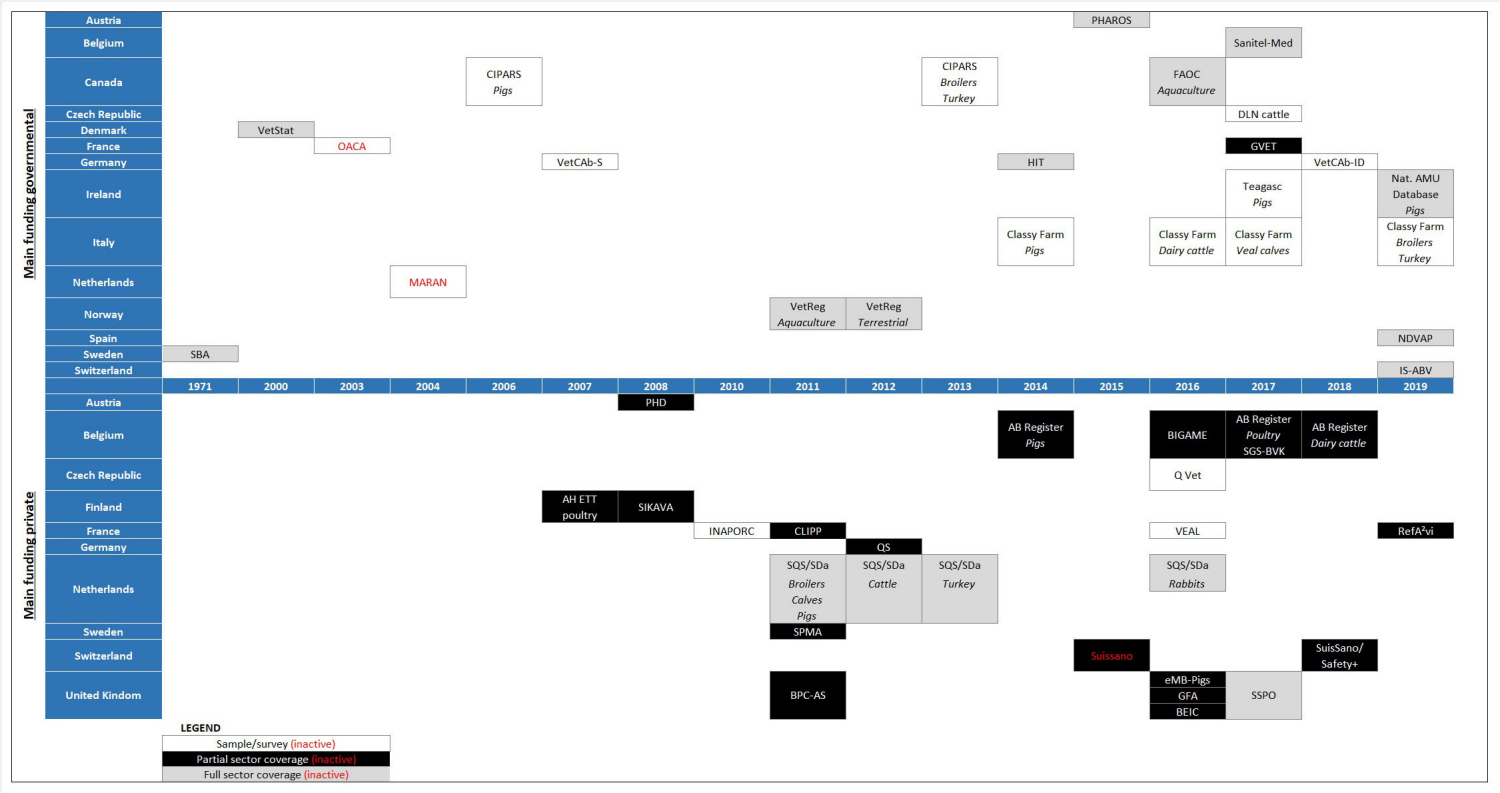
Data collection systems in each country, shown by start year of data collection and divided according to the coverage of the sectors included in each system (see “LEGEND”). A species below a system name is to indicate that the species was included in the system from that year on; no species indicated has no specific meaning—see further in the text for information on which species are covered in each system.

As of March 2020, 38 active farm-level AMU monitoring systems (further referred to as “systems”) from 16 countries were identified by the authors. Figure 1 lists all systems, including inactive systems, by year of official implementation and, if applicable, stratifies them by animal species. The oldest systems are those of the Swedish Board of Agriculture (SBA) and the Danish VetStat monitoring tool. Since 2010, many new systems have been set up in many countries and several existing systems were extended to additional animal species. As shown in Table 1, pigs and broilers are most frequently monitored, followed by calves and dairy cows.

Table 1Core characteristics of the currently existing systems for farm-level data collection of antimicrobial use data.

**Table:** 

**Country**^**a**^**–system**		**Collection**														
		**Animal type**^**b**^													**Input of AMU data^c^**	**Compulsory by^d^**
Austria	PHAROS	Pi	Da	Be	Ca	Br	La	Tu	Go	Sh					Vet	Legislation
Austria	PHD					Br	La	Tu							Vet	QAS
Belgium	AB Register	Pi	Da			Br	La	Tu							Vet·FM·PH	QAS
Belgium	BIGAME	Pi	Da	Be	Ca	Br	La		Go	Sh					Vet	QAS · voluntary
Belgium	Sanitel-Med	Pi			Ca	Br	La								Vet	Legislation
Belgium	SGS-BVK				Ca										Vet	QAS
Canada	CIPARS	Pi				Br		Tu							Farmer·Vet	NA: survey
Canada	FAOC											Fi			Farmer	Legislation
Czech Republic	DLN cattle		Da												Farmer·Vet	NA: voluntary
Czech Republic	Q VET pigs	Pi													Farmer	NA: voluntary
Denmark	VetStat	Pi	Da	Be	Ca	Br	La	Tu	Go	Sh		Fi		Ot: Mi	Vet·FM·PH	Legislation
Finland	AH ETT poultry					Br		Tu							Vet	NA: voluntary
Finland	SIKAVA	Pi													Farmer·Vet	QAS
France	CLIPP													Ot: Ra	Farmer·Vet·TN	NA: voluntary
France	GVET	Pi													Farmer	NA: voluntary
France	INAPORC	Pi													Farmer·Vet·FM·TN	NA: survey
France	RefA^2^vi					Br		Tu						Ot: ^*^^*^	Farmer·Vet	NA: voluntary
France	VEAL				Ca										Farmer·Vet	NA: voluntary
Germany	HIT	Pi		Be	Ca	Br		Tu							Farmer·Vet	Legislation
Germany	QS	Pi			Ca	Br		Tu						Ot: Du	Vet	QAS
Germany	VetCAb-ID	Pi	Da	Be	Ca	Br	La	Tu	Go	Sh	Ho	Fi	Pe	Ot	Not specified	Not specified
Germany	VetCAb(-S)	Pi	Da	Be	Ca	Br									Farmer·Vet	NA: survey
Ireland	Teagasc	Pi													TN	NA: survey
Ireland	Nat. DB pigs	Pi													Farmers	QAS
Italy	ClassyFarm	Pi	Da		Ca	Br	La	Tu							Researcher	NA: survey
Netherlands	SQS|SDa	Pi	Da	Be	Ca	Br		Tu						Ot: Ra	Vet	QAS
Netherlands	SDa^*^								Go	Sh	Ho		Pe	Ot: Mi	Vet	NA: survey
Norway	VetReg	Pi	Da	Be	Ca	Br	La	Tu	Go	Sh	Ho	Fi	Pe	Ot: De	Vet·FM·PH	Legislation
Spain	NDVAP	Pi	Da	Be	Ca	Br	La	Tu	Go	Sh	Ho				Vet	Legislation
Sweden	SBA	Pi	Da	Be	Ca	Br	La	Tu	Go	Sh	Ho	Fi		Ot: ^#^	Vet	Legislation
Sweden	SPMA					Br									Vet	QAS
Switzerland	IS ABV	Pi	Da	Be	Ca	Br	La	Tu	Go	Sh	Ho	Fi	Pe	Ot: Ra	Vet	Legislation
Switzerland	SuisSano|Safety +	Pi													Farmer	QAS
United Kingdom	BEIC						La								Farmer	QAS
United Kingdom	BPC-AS					Br		Tu						Ot: Du	Vet	PB
United Kingdom	eMB-Pigs	Pi													Farmer·Vet·FM	QAS
United Kingdom	GFA													Ot: Ga	Vet·FM	NA: voluntary
United Kingdom	SSPO											Fi			Vet	NA: voluntary

**Table:** 

**Country**^**a**^**–system**		**Analysis**^**e**^			**Benchmarking**^**f**^		**Reporting at farm level (Y/N)^**f**^**
		**Weight-based**	**Dose-based**	**Count-based**	**Y/N**	**Parties**	
Austria	PHAROS	–	DDDvet	–	Y	(Farmers) Vets	N
Austria	PHD	mg	–	Herds	Y	Farmers	Y
Belgium	AB Register	–	DDDA_bel_	–	Y	Farmers	Y
Belgium	BIGAME	mg/kg	DDDA_bel_	–	Y	Farmers	Y
Belgium	Sanitel-Med	mg/kg	DDDA_bel_	–	Y	Farmers·Vets	Y
Belgium	SGS-BVK	–	DDDA_bel_	–	Y	Farmers	Y
Canada	CIPARS	mg/PCU	DDDvetCA	Flocks/herds	N		Y
Canada	FAOC	mg			N		Y
Czech Republic	DLN cattle	mg	–	–	Y	Farmers	Y
Czech Republic	Q VET pigs	–	ADD	–	Y	Farmers	Y
Denmark	VetStat	–	ADD	–	Y	Farmers	Y
Finland	AH ETT poultry	–	–	Flocks	N		N
Finland	SIKAVA	–	–	–	(Y)		N
France	CLIPP	–	–	Days	Y	Farmers	Y
France	GVET	mg	UDD·UCD·DDD (vet) ·DCD(vet)	Days/animals	Y	Farmers	Y
France	INAPORC	mg	DDD(vet)·DCD (vet)	–	N		Y
France	RefA^2^vi	–	DDD_FR_·DCD_FR_	–	N		Y
France	VEAL	mg/animal	DCD_FR_	Days/animals	N		Y
Germany	HIT	–	–	Days/animals	Y	Farmers	Y
Germany	QS	–	–	Days/animals	Y	Farmers	Y
Germany	VetCAb-ID	–	–	Days/animals	N		N
Germany	VetCAb(-S)	–	–	Days/animals	N		Y
Ireland	Teagasc	mg/kg	–	–	Y	Farmers	Y
Ireland	Nat. DB pigs	mg/kg	–	–	N		Y
Italy	ClassyFarm	–	DDDA_IT_	–	Y	Farmers	Y
Netherlands	SQS|SDa	–	DDDA_NL_	–	Y	Farmers·Vets	Y
Netherlands	SDa^*^	–	DDDA_NL_	–	N		N
Norway	VetReg	mg	–	–	N		N
Spain	NDVAP	mg	–		N		N
Sweden	SBA	–	–	–	N		N
Sweden	SPMA	–	–	Flocks	N		N
Switzerland	IS ABV	–	PDD·DDDvet·DCDvet	Animals	(Y)	Farmers·Vets	(Y)
Switzerland	SuisSano|Safety +	–	DCDvet·DCD_CH_	Animals	Y	Farmers	Y
United Kingdom	BEIC	–	ADD	–	N		N
United Kingdom	BPC-AS	mg/kg	–	–	N		N
United Kingdom	eMB-Pigs	mg/kg	–	–	Y	Farmers	N
United Kingdom	GFA	mg	–	–	N		N
United Kingdom	SSPO	mg/kg	–	–	N		N

a *AT, Austria; BE, Belgium; CA, Canada; CH, Switzerland; CZ, the Czech Republic; DE, Germany; DK, Denmark; FI, Finland; FR, France; IE, Ireland; IT, Italy; NL, the Netherlands; NO, Norway; SE, Sweden; SP, Spain; UK, United Kingdom*.

b *Pi, pigs; Da, dairy cattle; Be, beef cattle; Ca, calves (veal and/or conventional); Br, broilers; La, laying hens; Tu, turkeys; Go, goats; Sh, sheep; Ho, horses; Fi, fish; Pe, pets; Ot, other, which can be De, (rein)deer; Du, ducks; Ga, game birds; Mi, mink; Ra, rabbits; in case of the SBA system in Sweden*,

# *stands for geese, ostriches, mink and reindeer*,

* *stands for all poultry production species including duck, guinea fowl, pigeon*.

c *Vet, veterinarian; FM, feed mills; PH, pharmacies; TN, technician*.

d *NA, not applicable; PB, Professional Body; QAS, quality assurance scheme*.

e *ADD, animal daily dose; DDDA_bel_, defined daily dose for animals as defined for Belgium; DCD_CH_, defined daily dose for animals as defined for Switzerland; DCD_IT_, defined course dose for animals as defined for Italy; DDD_FR_/DCD_FR_, defined daily/course dose for animals as defined for France; DDDA_NL_, defined daily dose for animals as defined for the Netherlands; DDDvet/DCDvet, defined daily/course dose for animals as defined by EMA (EMA, 2015); DDDvetCA, defined daily dose for animals as defined for Canada; PCU, population correction unit, as defined by EMA (EMA, 2011); PDD, prescribed daily dose; UCD, used course dose; UDD, used daily dose; DCD, defined course dose*.

f *Y/N, yes/no; (Y), planned for the (near) future*.

Three general features of the systems merit closer attention. The first is coverage, i.e., the proportion of the animal population included from the animal sector(s) targeted by a system. The identified systems can be divided into sample surveys (*N* = 11), partial coverage systems (*N* = 15), and full coverage systems (*N* = 12) (Figure 1). Full coverage systems aim to include all farms in an animal sector. Partial coverage systems include a substantial part of a sector, often on a non-random and possibly compulsory basis (e.g., adherence to a quality assurance scheme). For example, in the UK using the eMB-pigs system is a requirement under the farm assurance scheme “Red Tractor,” which represents 94% of UK pig farms. Sample surveys target a small and ideally random and representative sample of a sector. Alternatively, stratified sampling can be applied and, by weighting the results by stratum, a representative result for AMU in a sector can be obtained. Sample surveys are often intended to estimate the AMU within the sector and/or can be pilots to gain knowledge for establishing monitoring systems with broader coverage. For example, the MARAN data collection system in the Netherlands was used as the basis for the sector quality assurance systems that at present provide full coverage AMU monitoring (17). Participation in these systems can be a requirement for farmers to allow access to certain markets or customers. The German “VetCab” sample survey was the pilot for the implementation of the module for AMU in the “HIT” system and is now used to collect data for detailed analyses and to test options for methodological changes (18). The “HIT” system collects AMU data for almost all food-producing animal farms (although selection criteria on farm size are applied for major fattening livestock species). In France, the “INAPORC” surveys will continue to be used until the “GVET” monitoring system is more widely implemented (19). In the Czech Republic, pilot schemes are currently being used to plan for the establishment of broader systems later. Furthermore, in Italy data from the national electronic prescription system, which became mandatory in May 2019, is currently under processing and it will be used to improve 2020 pharmacosurveillance controls in pigs, cattle, and poultry. Despite their relatively limited scope, sample surveys can inform decision making, ideally when a representative sample is reached. In Canada, for example, data from a sample of sentinel farms was used to eliminate the preventive use of ceftiofur and fluoroquinolones in the poultry sector, as well as to reduce the use of some nationally defined medically important antimicrobials (20).

A second general aspect of the systems is their main funder, being either “private” or “governmental.” This is relevant for the “management role” in a system (i.e., who is operating the system?), as well as data ownership. Figure 1 shows that most systems are primarily government funded. Some systems, e.g., CLIPP in France, are jointly funded by private organizations and the government. Private organizations include quality assurance organizations, industry levy boards, or professional bodies.

When combining coverage and funding, it appears that the government is the main funder for most sample survey and full coverage systems. In contrast, private organizations are the main funders of almost all currently existing partial coverage systems. These systems generally target only farms that adhere to the respective quality assurance scheme and/or professional bodies.

The third general aspect, linked to coverage and funding, is the participation in the system, which can be “voluntary” or “compulsory” (Figure 1 and Table 1). Most systems with full coverage are compulsory, primarily by law/regulations. Several partial coverage systems are also compulsory as a requirement of quality assurance schemes. Caution should be exercised when interpreting data or extrapolating results from voluntary systems as these may represent the more conscientious and proactive farmers, and usually cannot be regarded as being representative of the population at large.

## Analysis of Key Components and Processes of Monitoring Systems for Farm-Level AMU

Four key components were distinguished for farm-level AMU monitoring systems: (1) data collection and quality control, (2) data analysis, (3) benchmarking, and (4) reporting.

### Data Collection and Quality Control

Data collection includes the process of entering raw data into the data collection system. Within the specific scope of monitoring farm-level AMU data, some aspects are particularly relevant.

At least two types of data need to be collected per identified farm: use data (also referred to as the “numerator,” see section “Data analysis”) and the animal population to standardize the use data (also referred to as the “denominator,” see section “Data analysis”). Use does not always constitute real administration of antimicrobials to animals; prescriptions, deliveries or sales at farm-level are often the only convenient data-source in practice. It is essential to record the time of use or date of delivery, in order to allocate the AMU to a certain time interval (see section “Benchmarking”).

Data collection can be automatic or manual. The former means the data are delivered digitally through software-linked data sources, e.g., from veterinary practice or farm management software, while the latter requires the data to be actively entered, e.g., by typing into an online interface, using prescription sheets, medicine books, as the data source. A pragmatic approach for data input is to offer both an automatic and a manual option. Automatic input will reduce the risk of typing errors and will significantly reduce the administrative burden for the parties providing the data. However, automatic input and transfer may require investment, training, and an adjustment of existing software. Manual input and transfer may therefore be offered as an alternative. In addition, if mistakes occur, manual correction of the automatic data input should be possible. When allowing manual retrospective corrections, a time-lock for accepting such changes should be considered, after which data entry cannot be altered by primary users. Allowing traceability of subjects uploading as well as altering the data is also considered useful. Data input in general (including “new” data) could be subjected to a time-lock, after e.g., a 1-year period. This may trigger users to frequently interact with the system and prevents continuous changes in the outcomes of the analyses, which is not desirable as the data might also be used for trend analysis and future calculations of AMU indicators. Note that a time-lock should not preclude correction of data errors. System administrators should always have the possibility to correct data errors, e.g., when obvious discrepancies are identified, and data quality could be considerably improved. In addition to using a time-lock, a logging system will be indispensable for enabling follow-up of the data-input. Data logs are useful for retrieving facts and figures for later analyses and for quality assurance.

To minimize the risk of data manipulation, careful consideration is required when determining which parties are authorized to alter the data, and which changes are allowed. Authority for alterations could, for example, be given only for data that the party has provided. Additionally, or alternatively, “party-over-party” checks could be required before accepting changes. For example, if the veterinarian provides the number of medication packages delivered and the farmer is permitted to make changes to that number, then the system might require final approval from the veterinarian. By imposing quality checks upon data input, the need to alter data at a later stage—and possible problems associated with such changes (e.g., changed benchmarking results)—might be avoided or minimized. This can be done by defining mandatory information to be included and running plausibility checks before the dataset can be submitted. Requiring active confirmation of a farmer that their data are correct might also be an option. This approach is particularly relevant for parties that actively register zero AMU, in order to distinguish true zero-users from farms with incorrect data or non-active farms. In the Belgian AB Register, farms that do not report any use over a certain period, without confirming this, are targeted by certified control agencies.

A quality check can also be implemented after sending the data. Standard quality measures should include plausibility checks of whether the reported variables are within the expected range, whether the identification numbers of core-variables are unique and whether the combination of categories is valid. In the latter case, for example, age groups, and disease groups should match the intended animal species, e.g., weaners should always be recorded in the animal category “pig” while the disease “goldfish ulcer disease” should only apply to “fish.” By processing the data into the anticipated result or by cross-checking with corresponding information in other databases or previous submissions, suspected mistakes can also be identified. After this checking step, validation should be requested, for example, by requiring additional (manual) confirmation or requiring input of the corrected data instead. For example, in the Netherlands, quality systems are notified of outliers by the Netherlands Veterinary Medicines Institute, and confirmation or correction is requested.

#### Use Data

For use data some specific aspects apply. Restricting and/or standardizing the options per input field will help to improve data quality. For example, in the Netherlands, a template is sent to the respective sector quality systems, which states the variables to be reported and their range. In the French “GVET” system, drop-down lists are included in the software and farmers select the veterinary medicinal products from a standardized list of all products authorized in France, where all medicines are linked with a unique identifier. They then choose one of the pre-set units of dosage (g/animal or g/100 kg of body weight for example) and there are also lists for other treatment characteristics (dates of administration, duration, indication, etc.). A similar approach is used in the Swiss system “IS-ABV.” The variables collected for the use data are dependent on the AMU indicator being calculated (see section “Data analysis”). As an example, the variables required in the Netherlands for the use data include the farm identification number, the delivery date of the antimicrobials, the European Article Number (EAN) of the antimicrobial and the number of packages delivered. The EAN is an identification number which is unique for every antimicrobial sold. In a database all antimicrobials used in livestock are recorded. This database also contains information on dosages (by animal species), administration routes, antimicrobial class, active ingredient(s), etc.

#### Animal Population Data

Depending on the AMU indicator being calculated, different types of animal population data can be collected (see section “Data analysis”). Within animal species, several animal categories can be distinguished to further refine AMU monitoring. For example, in Denmark and the Netherlands, three animal categories are defined for pigs: sows and piglets, weaned piglets and finishing pigs (21, 22). Animal population data per farm can be obtained internally or externally. Internally means that the data are collected specifically for the purpose of analyzing AMU data. For example, the monitoring system might require a farmer to provide the number of animals present (by production category) on his or her farm or the veterinarian might be required to record the number of animals on the farms visited. Regular inspections to obtain animal counts are also a possibility.

Externally acquired population data originate from sources established for purposes other than analyzing AMU data, typically not owned or managed by the AMU monitoring system, such as animal population data collected for epidemiological surveillance, for allocation of grants or for manure accounting. In the French GVET, for example, data from “GTE” is used, another French system whose goal is to yield technical-economic results to farmers. Data of produced biomass might also be obtained from slaughterhouses, which would have to register the number of animals slaughtered by farm. To improve data quality and for data management in general, it is advisable to minimize the number of “external” data sources. However, in many of the existing systems, animal population data are provided from external sources. In several systems, this is known to cause problems with retrieving updates or resolving problems in a timely manner. For example, in the Netherlands animal population data retrieved from the Central Board of Statistics were not always in line with animal population data collected by livestock sectors. Also updates in animal population data occurred after the annual report on AMU in the Dutch livestock sectors, leading to corrections in AMU figures after publication of the report. In Belgium, the SANITEL database for identification and registration of food producing animals does not contain animal numbers of all animal categories monitored in the AMU data collection systems. Furthermore, for several farms, SANITEL data are found to be incomplete or not up-to-date.

Additional data can be collected to allow for more detailed analysis and refined AMS. For example, use by animal production stage or type, the weight at treatment, indication(s) for treatment, and/or the prescribing veterinarian, type of administration (e.g., treatment, metaphylaxis, and prophylaxis) etc. However, the requested input should be of relevance to the analyses that will be carried out. Requesting too much or too detailed information will result in an unnecessarily high workload for the data provider and might lead to a subsequent lack of engagement.

It is also important to consider whether the data requires transformation prior to calculating AMU. For example, data on use of feed mixed with antimicrobials can be obtained directly by requesting the amount of premix delivered/mixed into the feed, or by requesting the amount of medicated feed delivered while also providing information on the concentration of premix in the medicated feed. Requesting untransformed data ensures a uniform calculation of AMU and is therefore preferred.

More practical information on data collection is provided in the guidelines (see AACTING website).

### Data Analysis

Data analysis is conducted to establish the farm-level AMU. Three important aspects of this calculation exist: selection of unit of measurement (UM), the animal population at risk (or denominator) and the indicator (Tables 1 and 2).

**Table 2:** Overview of count- and dose-based indicators calculated by different systems for analyzing AMU at farm-level.

**Country^a^**	**System(s)**	**Type^b^**	**Indicator^c^**	**Formula of indicator^c, d^**
Austria	PHAROS	Dose based	DDDvet/kg/year	mg AB used/DDDvet × n animals at risk × kg standard weight
	PHD	Count based	TH/UTH	n treated herds/n untreated herds
Belgium	All	Dose based	TD_100_	(mg AB used/DDDA_bel_ × kg animal at risk × n days at risk) × LA−factor × 100
	Sanitel-Med	Dose based^*^	Contract score	[(*% green ACU* ÷ 2) − (*% red ACU* ÷ 2) + 0, 5] × 100
Canada	CIPARS	Count based	pp TF|H	n treated flocks | herds/total n flocks | herds
		Dose based	DDDvetCA/PCU	$\left(\frac{\text{Milligrams active ingredient}/{\text{DDDvetCA}}_{\text{mg}/\text{kg}/\text{day}}}{\text{Total animals }\times \text{Standard weight at treatment }}\right)$
			DDDvetCA/1000 AD	$\left(\frac{\text{Milligrams active ingredient}/{\text{DDDvetCA}}_{\text{mg}/\text{kg}/\text{day}}}{\text{Total animals }\times \text{standard weight }\times \text{days at risk}}\right)\times 1,000$
Switzerland	IS ABV	Count based	ATI	n treated animals × n treatment days × n substances/n animals per year
		Dose based	Treatment intensity	(mg AB used/DDD_vet_ or DDD_CH_ × kg animal at risk × n days at risk) x 100
	SuisSano | Safety+	Count based	ATI	n treated animals × n treatment days × n substances/n animals per year^*LA Factor*^HPCIA Factor
		Dose based	DCDvet/animal/year	(mg AB used/DCD_vet_ × standard weight × n animals at risk per year)
			DCD_CH_/animal/year	(mg AB used/DCD_CH_ × standard weight × n animals at risk per year)
The Czech Republic	Q VET pigs	Dose based	ADD/100 animal-days	
Germany	HIT	Count based	Treatment frequency	n treated animals × n treatment days × n substances/n animals per day
	QS	Count based	Therapy index	n treated animals × n treatment days /total animal capacity
	VetCAb	Count based	Treatment frequency	n treated animals × n treatment days × n substances/total animal capacity
Denmark	VetStat	Dose based	ADD/100 animal-days	(mg AB used /technical daily dosage (ADD) × kg animal at risk × n days at risk) × 100
Finland	AH ETT poultry	Count based	pp TF	n treated flocks/total n flock
France	CLIPP	Count based	IFTA	$\overset{\text{z}}{\underset{\text{t}=1}{\sum }}{\left(\text{n treament days}\times \text{n substances}\right)}_{t}/\text{n days in reproduction cycle or rearing period}$ 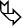 With t = the number of treatments
	GVET	Count based	Treatments/animal	$\overset{z}{\underset{t=1}{\sum }}$(n treated animals)_t_/n animals at risk 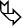 With t = the number of treatments
			Treatment days/animal	$\overset{z}{\underset{t=1}{\sum }}$(n treated animals × n treatment days)_t_/n animals at risk 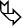 With t = the number of treatments
	GVET | INAPORC	Dose based	CD/animal	mg AB used/DCDA_FR_ × kg animals at riskn animals at risk
			DD/animal	mg AB used/DDDA_FR_ × kg animals at risk/n animals at risk
	RefA^2^vi	Dose based	DDD_FR_/kg slaughtered	mg AB used/DDD_FR_/kg animals slaughtered
			DCD_FR_/kg slaughtered	mg AB used/DCD_FR_/kg animals slaughtered
	VEAL	Count based	Treatments/animal	$\overset{\text{z}}{\underset{\text{t}=1}{\sum }}{\left(\text{n treated animals}\right)}_{\text{t}}/\text{n animals at risk}$ 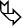 With t = the number of treatments
			Treatment days/animal	$\overset{\text{z}}{\underset{\text{t}=1}{\sum }}{\left(\text{n treated animals }\times \text{ n treatment days}\right)}_{\text{t}}/\text{n animals at risk}$ 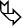 With t = the number of treatments
		Dose based	ALEA	mg AB used/DCDA_FR_ × n animals slaughtered × standard weight
Italy	ClassyFarm	Dose based	DCD_IT_/animal/period	mg AB used /DDDA_IT_ × kg animals at risk
The Netherlands	SQS/SDa	Dose based	DDDA_NL_/yr	kg treatable animals/kg animals at risk
		Der	VBI	AUC of ln-transformed ratio DDDA_nl_/yr and applicable thresholds over all 1-on-1-farms
Sweden	SPMA	Count based	pp TF	n treated flocks/total n flocks
The United Kingdom	BEIC	Count based	ADD/100 animal-days	
	BPC-AS	Mass based	mg/kg	mg AB used/kg animals at risk
	eMB-Pigs	Mass based	mg/kg	mg AB used/kg animals at risk
	SSPO	Mass based	mg/kg	mg AB used/kg animals at risk

a *AT, Austria; BE, Belgium; CA, Canada; CH, Switzerland; CZ, the Czech Republic; DE, Germany; DK, Denmark; FI, Finland; FR, France; IT, Italy; NL, the Netherlands; SE, Sweden; UK, United Kingdom*.

b* *Derived from (dose-based) farm-level benchmarking results*.

c *ADD, animal daily dose; ATI, (animal treatment index); ALEA, Animal Level of Exposure to Antimicrobials; CD, course dose; DD, daily dose; DCD_CH_, defined daily dose for animals as defined for Switzerland; DCD_IT_, defined daily dose for animals as defined for Italy; DDD_FR_/DCD_FR_, defined daily/course dose for animals as defined for France; DDDA_NL_, defined daily dose for animals as defined for the Netherlands; DDDvet/DCDvet, defined daily/course dose for animals as defined by EMA (EMA, 2015); DDDvetCA, defined daily dose for animals as defined for Canada; IFTA, Index of Frequency of Treatments with Antibiotics (number of treatment days related to rearing period); PCU, population correction unit, as defined by EMA (EMA, 2011); pp, proportion; TF|H, treated flocks | herds; UTH, untreated herds; VBI, Veterinary Benchmark Indicator*.

d *AB, active substance of an antibiotic; ACU, animal category unit, representing a single farm-level benchmarking result, with green being low use (= below the lower threshold as defined in the specific system) and red high use (above the higher threshold as defined in the specific system); AUC, area under the curve; DDDA_bel_, defined daily dose for animals defined at Belgian national level; LA-factor, long-acting factor; the technical daily dosage is based on the ADD principle, where each registered antimicrobial product in Denmark is assigned a specific dosage*.

#### Unit of Measurement

The UM is the unit in which the numerator is expressed. It can be mass-based, dose-based or count-based. Mass-based UMs express the numerator as milligrams, kilograms or tons (i.e., metric tons) of the active substance. Dose-based UMs express the numerator as the number of doses with several types being distinguished, e.g., defined daily dose animal (DDDA), used daily dose animal (UDDA), prescribed daily dose animal (PDDA), or defined course dose animal (DCDA) [(23–25); Table 2]. Typically, a dose-based UM is calculated from the amount of active ingredient using a mass-based UM. For example, if two 250 ml bottles of a medicinal product with one active antimicrobial substance at a concentration of 80 mg/ml have been used, a mass-based numerator indicates that 40,000 mg of active substance has been used. If these bottles have been used according to a prescription stating 8 mg/kg bodyweight per day, then a corresponding dose-based numerator indicates 5,000 PDDAs have been used (40,000/8 = 5,000).

For a count-based UM, the numerator can express the number of treatment days or treatment courses. Using the example above, if the medicinal product was used to treat a batch of 100 animals for 5 days, then the numerator would be 500 treatment days or 100 treatment courses. Hence, a count-based UM does not require data on the actual amount of antimicrobials used. It is worth noting that if, in the example given, each animal weighed ~10 kg and the prescribed dose of 8 mg/kg bodyweight per day was given, then this will correspond to a mass-based numerator of 40,000 mg of active substance used (100^*^10^*^5^*^8 = 40,000) and a dose-based numerator of 5,000 UDDAs (40,000/8 = 5,000). These examples illustrate that, although they have a different meaning, mass-, dose- and count-based UMs are interlinked.

The value, meaning, usefulness and complexity of defining and using (dose-based) UMs for the quantification of veterinary AMU have been addressed before (25). At EU level, (23) published a list of standardized Defined Daily Doses (DDDvet) and Defined Course Doses (DCDvet) for pigs, poultry and cattle, defined at the level of active ingredient and administration route and based on the Summary of Product Characteristics (SPC) for products authorized for that period in nine European countries (26). Table 1 illustrates the variety of UMs that are used. The choice of UM is highly dependent on the context (data availability, resources, surveillance objectives, etc.).

When working with mass-based UMs, farm-level AMU can appear to have improved by switching to medicinal products with a lower dose rate, whereas the level of animal exposure to antimicrobials may not have changed. Besides a lack of clarity around which species was treated, this is one of the main limitations of sales data. This is particularly true for some highest priority CIAs for human medicine, such as third and fourth generation cephalosporins, the use of which should be reduced in veterinary medicine.

When basing the analysis on the PDDA, automatic data input is only feasible if prescriptions are digitalized, which may require additional investment. Moreover, if the prescription does not specifically mention the dose in, for example, mg of active substance per kg bodyweight or mg of active substance per animal, the number of PDDA will require the data to be transformed first, particularly if an in-feed or in-water regimen is prescribed.

The UDDA can be calculated from administration data (treatment written/electronic treatment logbooks) and will, by definition, yield the most accurate reflection of the AMU on the farm. Depending on individual country regulations, working with the UDDA will often require the farmer/attending veterinarian to have a role in data input, either as a sole provider of the use data or for validating deliveries or prescriptions provided by another party (vet, feed-mill, etc.) based on what was actually used on farm. Count-based data, which are comparable to UDDA-based data in terms of outcome, can be determined from prescriptions, deliveries, as well as administration data and need input from farmers on what was really used for how many days and in how many animals.

If the intention is to be able to cross-check farm-level data with sales data on a national level, which are almost always mass-based, it must be possible to deduce the used mass of antimicrobials from the farm-level AMU data.

#### Denominator: The Animal Population

No matter what type of UM is used, the UM needs to be divided by a proxy for the targeted animal population to obtain comparable results. Different types of population data can be used, affecting the resulting indicator (25, 27).

The animal population is expressed as the mass of animals present over a defined period. This is either described as the biomass produced or the (average) mass of animals housed or a combination of both.

##### Biomass Produced

This can be based on the actual mass of meat produced or on the number of animals slaughtered multiplied by an estimated or standardized weight (standardized weights are discussed further in the text). For the calculation of farm-level AMU per year, it must be kept in mind that a denominator based on produced biomass does not reflect the true animal population at risk of antimicrobial treatment if multiple production cycles exist during that year. This is because slaughtered animals in high producing livestock sectors (such as swine and poultry), with multiple production cycles per year, have been at risk for antimicrobial treatment during their lifespan which is shorter than a year. This becomes evident when comparing AMU with species with longer production cycles (such as cattle) and/or countries where different farming practices apply (28). Therefore, biomass produced is not an appropriate denominator if the aim of a system is to calculate treatment incidences or frequencies at farm-level (estimating true exposure to antimicrobial treatment, see section about The Indicator). In contrast, it can be a useful denominator if, for example, the system aims for trend monitoring, e.g., for sector-level reduction targets, as exemplified by successful AMU reductions achieved in the UK (29). Also, if the aim is to compare AMU within a livestock sector per production cycle the biomass can be an appropriate denominator.

##### (Average) number or mass of animals at risk of treatment

The number of animals housed can be based on the maximum capacity of the barns (the maximum number of animals present on a farm), the number of animals present on average or the number of animals present at a given moment. This number of animals housed is then multiplied by an estimated or standardized weight in systems that use mass- and dose-based indicators. In contrast to produced biomass, the (average) number or mass of animals housed on farm is a suitable representation of the animal population at risk of antimicrobial treatment, hence, allows for calculation of treatment incidences or frequencies. The more the (average) number or mass of animals housed corresponds to the true number or mass of animals present at the time of treatment, the more accurate the calculation of exposure to AMU will be. To calculate this figure, capacity numbers are the least precise and accuracy increases when using stocking numbers, which need to be measured regularly.

Several options exist to determine the standardized or estimated weights that are used to establish the denominator (Estimated) liveweight at treatment will yield more precise results than an average weight (in a specific age category). For many animal categories, it is known that most antimicrobial treatments occur in the early stages of the animal's life (30–32). The estimated weight at treatment can be standardized, as suggested for example by EMA (EMA, ESVAC reflection paper 2013) and estimated standardized weights are applied in many countries (e.g., Belgium, the Netherlands, Denmark, United Kingdom). However, due to differences in production systems between countries and farms, animal weights at treatment might differ substantially and standardized weights may therefore be country- and livestock sector-specific. The estimated weight at treatment can also be determined based on age of the animal and a corresponding growth curve. This method will increase workload but will more accurately represent the animal liveweight at risk of antimicrobial treatment and will thus lead to more precise characterization of AMU.

For count-based systems, no weight is needed as either the UM is not reflecting the individual animals (i.e., number of farms using the drug is the UM) or the number of animals at risk is used in the calculation method. Kasabova et al. recently showed that differences in the weights used to calculate the population at risk have a substantial impact on the calculated AMU (31).

If the number of days at risk (i.e., the number of days each animal is present at the farm) is included in the denominator, i.e., the time interval during which AMU is assessed, the result will be a treatment incidence (33). The period at risk of treatment should correspond to the animal population at risk and to the period during which the numerator data are collected. As an example, if considering AMU over 1 year, the corresponding period at risk should be 1 year. In Denmark and Belgium 100 animal-days are used which is a proxy for the percentage of time an average animal is treated or the percentage of animals that are treated daily with one substance. In the Netherlands, the number of days an animal was treated in a year is calculated, which might be particularly useful for animals, such as dairy or beef cattle, with a longer production cycle.

#### The Indicator

The indicator is a technical unit used to quantify exposure to antimicrobials. Depending on the UM and the other parameters included in the calculation, different indicators will be obtained (23, 25, 27, 34). Tables 1, 2 illustrate the variety of indicators used in the different existing monitoring systems. Use of a mass-, dose-, or count-based UM will, respectively, lead to a mass-based indicator, such as mg/kg biomass or mg/PCU (population correction unit), a dose-based indicator, such as number of DDDA/1,000 animals produced or number of DDDA/animal year, or a count-based indicator, such as the treatment frequency. As indicated in Table 1 most indicators included here are dose-based, a minority are mass-based, and some count-based indicators are calculated.

The choice of the indicator impacts on the interpretation of AMU monitoring results. However, deciding on which AMU indicator to use can be complex, given the range of existing options. The guidelines on the AACTING website highlight various aspects to consider when deciding which indicator to use for AMU monitoring.

### Benchmarking

Benchmarking of AMU refers to the comparison of a party's AMU with the AMU of similar parties (the reference population), given that AMU for all parties is quantified in a comparable manner. To the authors' knowledge, benchmarking is currently carried out—or planned to be carried out as soon as good quality data are available and a methodology is developed—in 12 countries, encompassing 20 AMU monitoring systems (Tables 1, 2). Most existing AMU monitoring systems benchmark farmers; three systems benchmark veterinarians (Table 1).

Benchmarking is performed with a certain frequency (for example, twice a year) and takes AMU within a certain time interval (for example, the preceding year) into consideration. The shorter the production cycle of the animal species or animal category for which the AMU is benchmarked, the greater the relevance of a high benchmarking frequency. The time interval should depend on the expected influence of recurring events (for example, seasonal influences) and needs to find a balance between allowing for frequent reporting of the benchmarking indicator (short time interval) and obtaining a longer-term view of AMU (long time interval). A longer time interval may be useful to achieve more sustainable use practices but could lead to issues with perceived “fairness” or “relevance” of the score if antimicrobial use distant in time still impacts the current benchmarking result.

Various aspects of AMU can be benchmarked using different indicators. A starting point at farm-level is the total AMU on the farm (per species or, if different age/weight categories of a species are present, per production stage). Furthermore, various qualitative aspects of AMU can be benchmarked, e.g., the use of certain classes or categories of (critically important) antimicrobials, the type of treatment [e.g., veterinary medical use vs. non-veterinary medical use (35)], the route of administration (e.g., oral, parenteral, and intramammary). It might be advisable not to benchmark too many aspects, as multiple benchmarking results for a single species (category) might become confusing and end up being counterproductive, especially if the results appear contradictory.

The reference population can be based on geography (e.g., country, region), economic traits (e.g., sector, quality assurance scheme), animal traits (e.g., species, age/weight category), or simply on selection criteria and the willingness of parties to co-operate (e.g., in a research study). In practice, combinations often occur, e.g., benchmarking within a group of farms adhering to a certain quality assurance scheme and raising fattening pigs. In systems with only partial coverage, special attention should be paid to defining the reference group to avoid drawing conclusions based on a few farms or particular farm types.

The more relevant the chosen reference group, the more practically useful benchmarking will be. For instance, in veal calves, it might be decided to benchmark among veal calf farms in general. Making a further distinction between production stages/types, for example starters and finishers or rosé and white veal farms (if applicable) might add important nuances to the result (36). However, defining too many reference groups, each with their own thresholds for (un)acceptable use, can be counterproductive. For example, if reference groups are chosen according to farm management characteristics (e.g., weaning age in pigs, breed), high use caused by using a vulnerable breed that is more prone to infectious diseases might be deemed acceptable by the benchmarking system.

Two types of benchmarking can be distinguished: “dynamic” and “fixed” benchmarking. We define “dynamic benchmarking” as a methodology where the benchmarking result depends on the distribution of AMU in the reference population. Ranking a party within the reference population (e.g., farm X is at the 40th percentile) is one form of dynamic benchmarking. Another is using one or more threshold values derived from the distribution of AMU in the reference population (e.g., the median and the 75th percentile) and categorizing farms relative to these thresholds. In contrast, “fixed benchmarking” uses reference values that do not (always) reflect the distribution of AMU in the reference population. Such threshold value(s) are typically set for a long(er) period. Generally, the distribution of AMU in the reference population is used to initially set or adjust the fixed threshold(s). However, “politically” motivated thresholds are also used, i.e., thresholds that are not related to the current distribution of use but state a fixed reduction target, e.g., reduction by 20% over a certain period.

Dynamic benchmarking is applied, for example, in Belgium and Germany, and the systems involved apply two threshold values. In Denmark, fixed benchmarking with one threshold is applied. This is also the case in the Netherlands, the latter having evolved recently from a fixed benchmarking method using two threshold values to now using only one. In Belgium, the pig sector has recently started working with fixed benchmarking with two threshold values.

A farm-level AMU distribution is in most cases right-skewed, with a long tail toward the high-user end of the distribution (22). For this reason, using the arithmetic mean AMU as a reference or threshold value may not be ideal. Therefore, the median value is often used as the lower threshold, for example, in Belgium and Germany. As the upper threshold, the third quartile (75th percentile) is used in Germany and the 90th percentile in Belgium.

Fixed benchmarking does not mean the threshold values will never change. Adaptations of thresholds according to changes in use or changes in policy are advisable. As described above, the Netherlands recently revised their threshold values, as the original benchmark values were no longer deemed to provide enough incentive to further reduce AMU in several livestock sectors (37). In Denmark, the threshold values have been revised five times since they were launched in 2010 (21). Furthermore, a differentiated benchmarking strategy was implemented in 2016, with certain antimicrobial classes being weighted with higher factors in the quantification of AMU in this country.

An advantage of dynamic benchmarking is that it is more difficult for the benchmarked parties to circumvent prudent usage policies by strategic changes in their AMU to comply with the threshold values. Dynamic values ensure that constant pressure is sustained for parties to keep reducing their AMU. However, this might have a discouraging effect, as reducing AMU does not necessarily mean that a party will reach a level below the threshold, because the threshold may have been lowered as well. A challenge of dynamic benchmarking is that data validation needs to be finalized and the data need to be fixed before benchmarking is applied. This avoids the reference group changing over the course of the process, as parties with incorrect data may be excluded (and later re-included) from the reference group during benchmarking periods, which would result in different benchmarking thresholds. This shows that working with dynamic thresholds can be technically harder and analyzing trends is more challenging. As a result, using dynamic thresholds in the design and communication of antimicrobial policy measures is generally more complex.

When applying fixed benchmarking, two thresholds might be set. With two thresholds, three zones are created: a zone with “desirable or accepted use,” with AMU below the lower threshold; an “attention” zone, with AMU between the lower and the upper threshold and an “action” zone, with AMU above the upper threshold (see Supplementary Figures 1, 2). This approach allows the system to focus on the highest users of antimicrobials, yet allows the group of “elevated attention” users to be identified and alerted.

Ultimately, using only one fixed threshold value (per animal species or category) is the most straightforward approach (i.e., the level of AMU is categorized as acceptable or not) and administratively and technically the least complex. As shown in the Netherlands and in Denmark, this is particularly advisable if the distribution of the AMU in the reference population is generally low and less right-skewed. Adaptation to such a threshold will in this case not be such a problem. If this is not the case an “attention zone” might be useful, with farms within this zone receiving a warning but without warranting immediate action. The consequences of setting the level of a threshold, in terms of number of farms exceeding the threshold(s) and the corresponding workload, should be kept in mind.

For the purpose of AMS and to encourage behavioral changes, benchmarking is particularly relevant if the outcome has consequences for the benchmarked party. Across countries, different risk management measures or interventions are triggered when thresholds are exceeded. Examples include the requirement to draw up action plans to reduce AMU, detailing additional measures that will be taken to reduce disease incidence, additional veterinary or inspection visits, compulsory advice to be obtained from an external party, fines, or exclusion from quality schemes. Furthermore, more corrective measures could be foreseen if AMU levels remain above a certain threshold for an extended period. Ultimately, animals or their products could be considered unfit to enter the food chain. On the other hand, parties with prudent and responsible use might also be rewarded. Such positive consequences might be social, e.g., making the good results of farms visible through certification or other “signs of recognition,” or financial, through for example a bonus for good results. However, very low or zero use should be validated, as use might not have been correctly reported. It is important to note that the goal should be to use antimicrobials prudently, which should lead to an overall reduction in AMU. This should be predominantly achieved by only using antimicrobials when necessary to ensure animal welfare.

Parties should be granted adequate time between receiving a benchmarking result and the deadline for subsequently achieving a reduction in AMU. For example, if benchmarking results are available every trimester, it is unfeasible to require a reduced AMU by the next reporting cycle when a single production cycle lasts 6 months. In the interests of fairness, it should also be possible to appeal against a result for a short period after receiving the benchmarking result.

The success of benchmarking in terms of AMS will increase if the analysis and benchmarking methodology are transparent and clearly communicated to the affected parties.

Considering the responsibility of veterinarians for prescribing antimicrobials and therefore directly influencing AMU in animals, benchmarking veterinarians is an important option from an AMS perspective. Depending on the country and its legislation, it is not necessarily the AMU of a veterinarian that is being benchmarked but rather their “antimicrobial prescribing behavior.” For convenience, we will denote this as “AMU of veterinarians” for the remainder of this paper. Similar definitions and principles apply for benchmarking the AMU of veterinarians as outlined above for benchmarking the AMU of farmers. However, benchmarking the AMU of veterinarians is more complex. The benchmarking score of a veterinarian needs to be calculated from the AMU of multiple farms. This can be challenging, as differences exist between veterinarians in terms of the number and characteristics of the farms he or she is responsible for. In addition, compared to benchmarking farmers, who are (legally) responsible for actions taken regarding the health of animals on their farm, it is more difficult to make veterinarians feel responsible for the AMU on a farm if it is being serviced by other vets, i.e., if there is no strict one-to-one relationship.

Usage results may be biased due to different health status and size of farms serviced by a practice. The Netherlands was the first country to adopt benchmarking of veterinarians. The Dutch veterinary benchmark indicator (VBI) calculates the probability of the farms, for which a certain veterinarian is responsible, falling within the action zone [i.e., above a certain AMU threshold; (38, 39)]. This methodology is now under revision (22, 37). In 2019, the Belgian “Sanitel-Med” system launched its benchmarking of veterinarians. Two scores are applied: (1) a contract score, expressing, for farms on which the veterinarian is the designated “herd vet,” the distribution of animal categories falling within the “low,” “attention,” and “high” AMU zones in the benchmarking at farm-level; (2) a management score. The latter expresses the proportion of the total AMU of a veterinarian that was used at farms where the veterinarian was not the designated herd veterinarian. Austria also recently implemented a benchmarking system for veterinarians. Switzerland and France have tools that may be used in the future to apply benchmarking of veterinarians, but the protocols are not yet established and implemented.

Benchmarking of AMU of farms and/or veterinarians can be a powerful tool in reducing AMU as shown in Denmark, Germany and the Netherlands (21, 22, 40).

### Reporting

Within the scope of this review, reporting refers to the process of providing feedback about farm-level AMU to the farmer or other parties. Such a process is critical, especially in terms of AMS. Of the four processes discussed here, reporting is the most subjective one, and needs to be adapted to the target audience. Consequently, discussing the value of different reporting formats is beyond the scope of this paper. Examples are provided only to illustrate some of the possible options.

Three types of target groups can be broadly distinguished: farmers and veterinarians, regulators and stakeholders (government, industry, sector organizations, farm assurance schemes) and the general public/consumers. In terms of AMS, farmers and veterinarians are the group most suited for receiving individual benchmarking results. Summarizing reports, typically not containing individual results, are more useful for a wider audience. The latter reports focus on general trends, achieving policy targets, comparing AMU among different animal species, categories, or other subgroups, etc. Several countries have been publishing reports of antimicrobial sales data for many years, increasingly including AMU data and sometimes AMR data as well [examples from countries in the AACTING network (21, 22, 29, 41–44)]. Reports for regulators can fall in either of the two categories, i.e., the individual party and the anonymous summary reports. In Belgium, yearly summarizing reports, without individual farm results, are also made available for some quality assurance schemes. Regardless of the purpose of data collection and the report type, the ownership of the data and their confidentiality must be defined and strictly adhered to. Any communication of results to third parties must be approved by the data owner. Summary results such as general trends by sector can, however, generally be published. Data ownership by the government is another option.

In terms of policy making and auditing, reporting of farm-specific results should be periodic, i.e., at pre-defined times and for all farms. In the Netherlands, the “SDa” reports analyses on AMU to regulators, policy makers and the general public, while the quality systems report, in parallel, to their farmer members. Private quality assurance systems of the monitored livestock sectors report results and provide feedback to individual farmers about their AMU. Broiler farmers, for example, receive a report every 3 months in which several aspects of their AMU are compared to sector averages. If a farmer's AMU is considered too high, measures are required to reduce use. In this respect, it might be interesting to additionally provide means of evaluating the result in real-time (for example through an online portal), especially if the frequency of periodic reporting is low (for example once a year) and the animals' replacement rate is relatively high.

Reporting will have the greatest effect when the (quantitative) information is given in relation to a reference population, hence as a benchmarking result. Showing a specific farm's AMU within a distribution of AMU within that sector/animal category makes comparisons easier and more illustrative for farmers (Supplementary Figure 1). Moreover, this could lead to “social pressure,” known to be one of the five cues for changing human behavior (45). Most systems that perform benchmarking also report the results to all the parties involved (Table 1).

In addition to the quantitative use results, reports may contain guiding information, such as directives, data on farm trends and more detailed qualitative analyses, creating more insight into farm-level AMU (Supplementary Figure 2). In the Netherlands, antimicrobials are classified as either first, second or third choice antimicrobials, depending on resistance inducing effects and importance to human health. A similar system exists in Belgium, using color codes (yellow, orange, and red antimicrobial classes, respectively). The use of different choices of antimicrobials is also reported to individual farmers. Other aspects of interest to report to farmers could include use of group treatments, use of medicated feed containing antimicrobials, use of intramammary tubes, age at treatment, indication for treatment, etc.

Ultimately sustained behavioral changes in veterinarians and farmers are needed to establish prudent AMU. Invoking behavioral changes is complex and dependent on the target audience. Speksnijder and Wagenaar described how sociopsychological models can provide insight into a farmers' and veterinarians' behavior regarding AMU practices and how behavioral changes can be motivated (46). Several studies report that veterinarians perceive their main role as a service provider, they do not feel a demand from their farmers for advice (47–49). Insights obtained from sociopsychological models might also support veterinarians in their advisory role toward improving a farmer's management (46). Communicating best practices to farmers as well as veterinarians might be a helpful tool to encourage farmers and veterinarians to reduce AMU (50).

## Discussion

This overview shows a very large variety across systems, especially at the level of analysis (choice of numerator, denominator and indicator). As AMU reduction figures for several countries have proven the principle of implementing farm level monitoring might be a more defining factor for success than the actual methods used. Furthermore, each system has its limitations, perhaps not so much in design as in execution: the results (calculated AMU) largely depend on the degree to which the data were provided to the system(s) correctly and in a timely manner. Hence, it could be argued that aiming for harmonization across systems is not only unfeasible but also unnecessary.

Nonetheless, a desire to compare, essentially between similar sectors in different countries, does exist (13). In this regard, it is remarkable that almost all systems deploying farm-level benchmarking in partial or full coverage systems use an indicator that reflects the (true) exposure of animals to AMU, by calculating a treatment incidence or treatment frequency (Table 1). It is for this reason that an indicator reflecting the number of treatment days out of 100 days present on the farm has been specifically brought forward in the AACTING guidelines, published in the scope of the AACTING project, and with the aim of assisting parties in setting up systems for monitoring of farm-level AMU (see www.aacting.org/aacting-guidelines). Although, as noted above, it is clear that various other indicators are just as valuable, the value of this particular indicator lies in the fact that its outcome is a meaningful parameter for the farmer and veterinarian, and that it is a flexible indicator, which makes it possible to calculate back to mg/kg or to transform to a treatment frequency (when using the UDD instead of the DDD). As a secondary effect, these guidelines might be a step toward more harmonized approaches for farm-level AMU monitoring. It should be recognized that there is potential for more harmonization. Currently, a lack of harmonization is one of the factors that limits comparisons of AMU between farms within countries and comparisons of farm-level AMU data between countries, even though such comparisons are important for improving AMU practices (25, 51). More harmonized approaches to monitoring AMU at farm-level will improve understanding, communication, and sharing knowledge regarding AMU monitoring and benchmarking. Yet, even if using harmonized usage indicators, comparing systems in different countries might be seriously limited by variations in health and husbandry conditions, for example, the production cycle length of animals, availability of medical products, and regulations. It remains the responsibility of the parties aiming for, or performing any, comparisons of systems to carefully address the differences between them, which will not be resolved by choosing a uniform indicator.

Apart from bringing forward the usefulness of a specific type of indicator for monitoring farm-level AMU, the AACTING guidelines had the aim of providing useful information as how to organize the data collection, what to take in consideration when choosing a methodology for analysis, principles of benchmarking, and critical factors for reporting. The rationale for many of the points raised in the guidelines is provided in this overview. It is therefore advised to jointly consider both documents.

In conclusion, quantifying AMU at the farm-level represents an important step toward AMS, as mirrored in the ESVAC vision for 2016–2020. Recently, an increase has been observed in the number of countries developing AMU quantifying systems. An important aim of the AACTING consortium is to provide information about existing systems and support in designing new systems or revising existing systems. In this paper, we investigated the necessary components of such systems and described the available options, taking into consideration a selection of systems that currently exist. Based on this overview and the combined expertise of the international authors, we proposed a set of guidelines on the collection, analysis, benchmarking and reporting of AMU. These guidelines represent a relevant tool for entities planning to establish new farm-level systems for quantifying AMU and shall ideally contribute to the standardization of methodologies.

## Author Contributions

WV drafted a first version of the manuscript. PS, MF, KF, WO, AA, CCa, BB, VD, CCh, AH, AK, RM, GA, FS, KS, CM, IG, FB, LP, CF, LC, EM, LJ, MS, JP, SB, DH, and JD give their initial comments on the first version. PS, WV, MF, DH, and JD reworked the manuscript to all its subsequent versions, with the others authors contributing equally in subsequent revisions, up to the final version. WV and PS have contributed equally to the final version and share first authorship. All authors contributed to the article and approved the submitted version.

## Conflict of Interest

The authors declare that the research was conducted in the absence of any commercial or financial relationships that could be construed as a potential conflict of interest.

## Footnotes

^1^www.aacting.org

## Definitions and Abbreviations

AMU: Antimicrobial use.
AMR: Antimicrobial resistance; the ability of microorganisms of certain species to survive or even to grow in the presence of a given concentration of an antimicrobial agent that is usually sufficient to inhibit or kill microorganisms of the same species (52). Only acquired resistance is considered within the scope of this publication.
Benchmarking: The comparison of a party's AMU with the AMU of similar parties (the reference population), given that AMU for all parties is quantified in a comparable manner.
AMS: Antimicrobial stewardship; AMS is aiming for prudent and appropriate AMU, which is defined by the World Health Organization (WHO) as “use of antimicrobials, which maximizes therapeutic effect and minimizes the development of antimicrobial resistance”; according to the OIE, AMS consists of “a set of practical measures and recommendations intended to prevent and/or reduce the selection pressure of antimicrobial-resistant bacteria in animals” (53). This also includes preventive measures to avoid antimicrobial use.
CIA: Critically important antimicrobials as defined by the WHO. These antimicrobials are considered critically important for human health according to two main specific criteria (54).
Party: Any person, group of persons or organization involved in any of the processes of data collection, analysis, benchmarking, etc., such as farmers, herd managers, veterinarians, pharmacies, researchers, and system administrator(s).
DDDA: Defined daily dose animal; the assumed average dose of an antimicrobial per kg animal body weight by animal species. The EU standard is the DDDvet (24).
DCDA: Defined course dose animal; the assumed average dose of an antimicrobial per kg animal body weight per treatment course for a specific animal species. The EU standard is the DCDvet (EMA, 2015).
UDDA: Used daily dose animal; the actual dose used of an antimicrobial per kg animal body weight of active substance for a specific animal species.
PDDA: Prescribed daily dose animal; the dose of an antimicrobial (or active substance) that is on average prescribed per kg animal body weight per day for a specific animal species.
UM: Unit of measurement; the unit in which the amount of antimicrobials used is expressed.
Indicator: The parameter quantifying use of antimicrobials, generally calculated as “number of units of measurements” (the numerator) divided by the “animal population” to standardize the use data (the denominator).
