# Supplementary material for: Monitoring of Farm-Level Antimicrobial Use to Guide Stewardship: Overview of Existing Systems and Analysis of Key Components and Processes
Source: Front Vet Sci. 2020 Aug 21;7:540. doi: 10.3389/fvets.2020.00540 (PMC7475698; doi:10.3389/fvets.2020.00540)
Supplement: Supplementary file 1 [file Data_Sheet_1.docx]

Supplementary Material

## Supplementary Figures


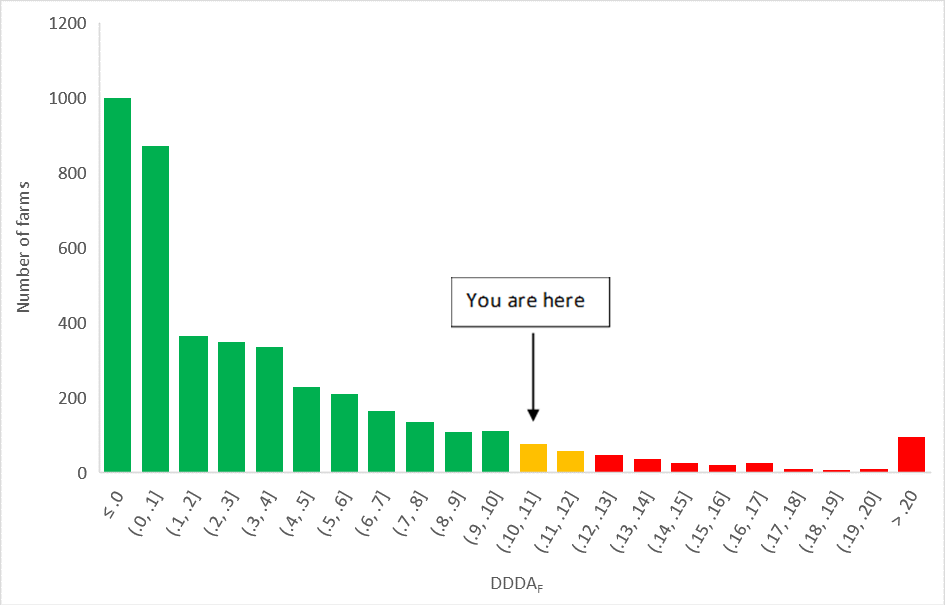
***
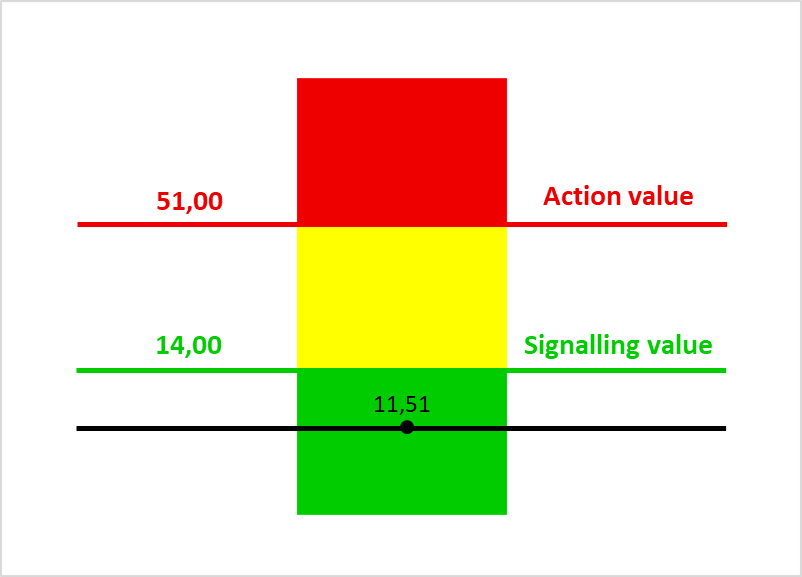
***

**B**


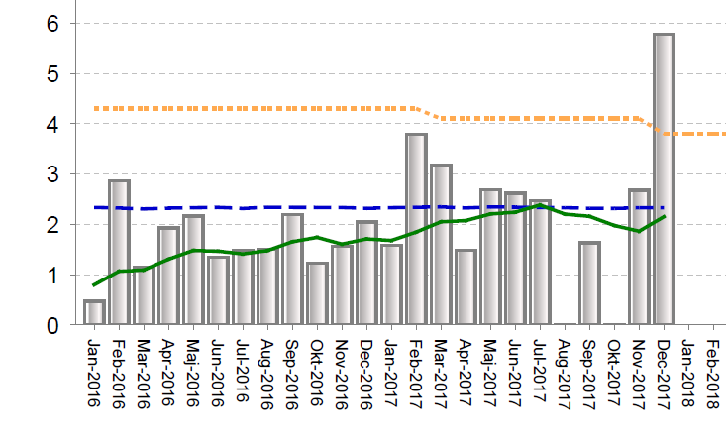


**A**

**C**

**Supplementary Figure 1.** Different reporting examples of benchmarking results of AMU at farm (or animal category) level, from the Netherlands (A), Belgium (B) and Denmark (C). The (quantitative) information is given in relation to a distribution of AMU in that sector/animal category reference population.


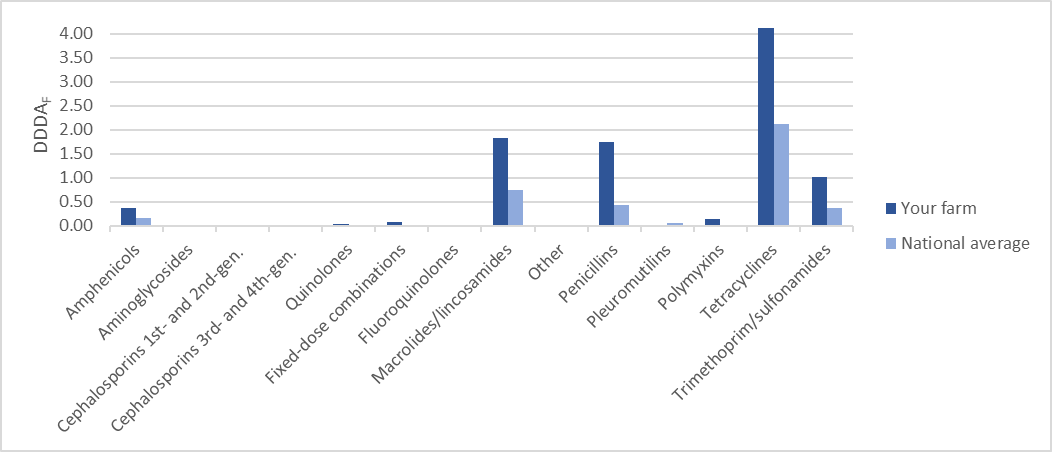


**A**

***
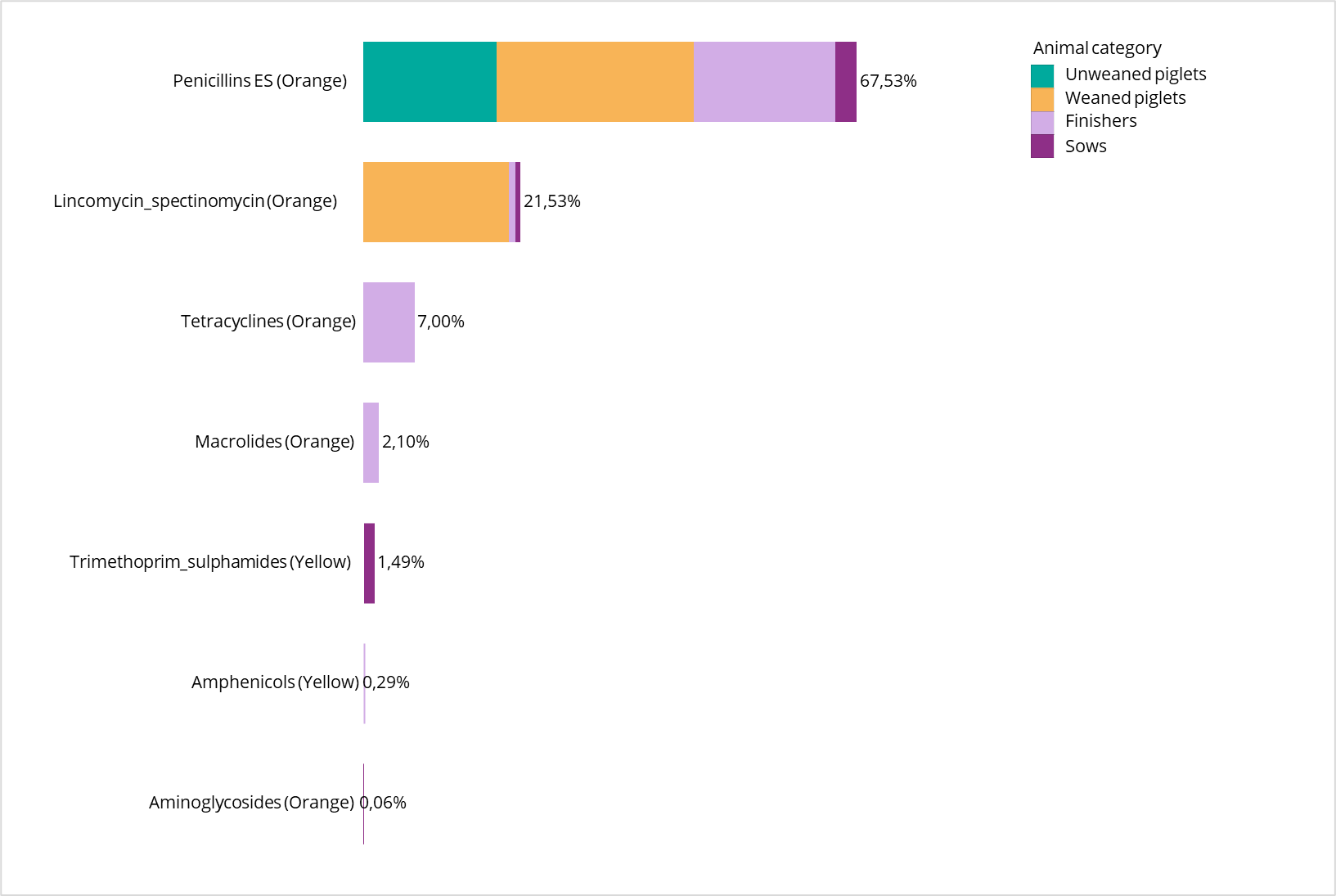
***

**B**

**Supplementary Figure 2.** Different reporting examples of more detailed qualitative analyses of AMU at farm (or animal category) level, from the Netherlands (A) and Belgium (B). For the Netherlands, AMU per antimicrobial class is compared between a pig finisher farm and the national average.
